# A Hybrid 3D Printed Hand Prosthesis Prototype Based on sEMG and a Fully Embedded Computer Vision System

**DOI:** 10.3389/fnbot.2021.751282

**Published:** 2022-01-24

**Authors:** Maria Claudia F. Castro, Wellington C. Pinheiro, Glauco Rigolin

**Affiliations:** ^1^Electrical Engineering Department, Centro Universitário FEI, São Bernardo do Cambo, Brazil; ^2^Mechanical Engineering Department, Centro Universitário FEI, São Bernardo do Cambo, Brazil

**Keywords:** hand prosthesis, computer vision, myoelectric signal, convolutional neural network, 3D printed

## Abstract

This study presents a new approach for an sEMG hand prosthesis based on a 3D printed model with a fully embedded computer vision (CV) system in a hybrid version. A modified 5-layer Smaller Visual Geometry Group (VGG) convolutional neural network (CNN), running on a Raspberry Pi 3 microcomputer connected to a webcam, recognizes the shape of daily use objects, and defines the pattern of the prosthetic grasp/gesture among five classes: Palmar Neutral, Palmar Pronated, Tripod Pinch, Key Grasp, and Index Finger Extension. Using the Myoware board and a finite state machine, the user's intention, depicted by a myoelectric signal, starts the process, photographing the object, proceeding to the grasp/gesture classification, and commands the prosthetic motors to execute the movements. Keras software was used as an application programming interface and TensorFlow as numerical computing software. The proposed system obtained 99% accuracy, 97% sensitivity, and 99% specificity, showing that the CV system is a promising technology to assist the definition of the grasp pattern in prosthetic devices.

## 1. Introduction

The main function of the human hand and upper limb is to grasp and manipulate objects. Thus, this loss affects the ability of the amputees to carry out activities of daily living, leading to a significant impact on their independence and quality of life. Today there are some sophisticated commercial robot hands available in the market, such as iLimb Ultra and iLimb Quantum by Össur (2021b,a) and Bebionic Hand and Michelangelo Prosthetic Hand by Ottobock (2021a,b). However, the need for affordable prosthetic devices has driven the development of 3D printing systems in order to enable their use by a greater number of people. OpenBionics (2021) and InMoov (2021) are open-source initiatives for the development of affordable, lightweight, and modular myoelectric prosthetic devices that can be easily reproduced with commercially available materials.

The simple structural design of DC or servo motor wired-driven mechanisms controlled by a surface myoelectric signal (sEMG) became popular (Abarca et al., 2019; Ku et al., 2019; Sureshbabu et al., 2019; Mohammadi et al., 2020; Wahit et al., 2020; Khan et al., 2021). The sEMG control can be as simple as an on-off control scheme, proportional where movement velocity depends on the muscle contraction intensity, and even by pattern recognition, which classifies the sEMG into grip pattern classes, have also been used (Geethanjali, 2016). However, while in the former, the number of possible grasp patterns is limited, the success of the latter in clinical applications depends on the users' ability to generate distinct commands in a reproducible manner, being difficult to amputees. Users may get frustrated and stop using the prosthesis quickly (Scheme and Englehart, 2011; Jiang et al., 2012; Palermo et al., 2017; Zhai et al., 2017).

Computer vision (CV) can help the system to better understand the visual world, simulating tasks in the same way that human vision does. Algorithms that give a visual perception to the system can, for example, identify the type of object to be picked up and associate it with the appropriate grasp to be performed.

Dosen et al. (2010) and Dosen and Popovic (2010) proposed a simple scheme, using a web camera and an ultrasound distance sensor. After processing grayscale images at a resolution of 320 × 240 pixels, using LabView 2009 running on a standard laptop (dual-core 2 GHz Pentium), they extracted parameters such as the lengths of the long and short axes and the orientation angle of the long axis concerning the horizontal axis of the image plane. Based on the object size and series of rules, one of four grasp types was selected, being pinch, key grasp, palmar, or spherical.

The InMoov hand was modified by Sidher and Shen (2017) enabling the opposability of the thumb and the introduction of two cameras and proximity sensors on the palm, allowing object detection and automatic grasp definition. Two Raspberry Pi 3 (RPI3) were used to control the cameras, and the control of the servos was achieved by an Arduino Mega, all controlled by a Matlab algorithm running on a PC. However, in Sidher (2017) tests were made only with geometrical objects with a tripod grasp.

DeGol et al. (2016) proposed the inclusion of a CV based on a convolutional neural network (CNN) with an architecture based on the VGG-VeryDeep-16 in a prosthesis for the automatic selection of the grasp to be performed into five classes: power grasp, pinch, tripod pinch, tool, and key grasps. The system achieved 93.2% accuracy running on an NVIDIA Tegra GPU for image processing.

In Andrade et al. (2017), the image captured by an embedded camera was processed on an external server through the Inception-v3 and Tensorflow, and the suggested grasp returned to the local processing unit, an RPI3. The user could accept or cancel the result. If accepted, the resulting grasp pattern went to the V-rep simulator system, which had 14 grasp pattern possibilities: relaxed hand, active index finger, tool, abducted thumb, index flexion, hook, key grasp, use with a computer mouse, open palm, pinch, power, precision gripper opening, precision gripper closing, and tripod pinch. The Myo armband was used to trigger a state machine to take a picture, validate a proposed grasp, ask for another grasp, or cancel an operation, using “wave in,” “wave out,” and “fist” contractions.

Another study that also used a CV system with a two-layer CNN to classify objects into their respective grasp patterns was presented by Ghazaei et al. (2017). Over 500 objects from Amsterdam and Newcastle Grasp Libraries were categorized into four grasp classes, named: pinch, tripod, palmar wrist neutral, and palmar wrist pronated. The classification accuracy in the offline tests reached 75%. In a real-time experiment with a set of the novel as well as seen but randomly-rotated objects, the system achieved an overall score of 84%, implemented in MATLAB on a Lenovo laptop with an Intel Core i7-4559U CPU (2.10 GHz).

A multimodal system was proposed by Shi et al. (2019) combining eye tracking, CV, sEMG, and an Inertial Measurement Unit (IMU) integrated into the HIT AID Hand prosthetic device. The Kinect 2.0 (Microsoft, USA), a 3D Camera, collected color, depth, and infrared scene images from the user's perspective. The selection of the target objects was through gazing (Eye-Tracking), and the grasp pattern was defined among four based on a convolutional network model. The user controls the prosthesis in collaboration with both sEMG and IMU.

A more sophisticated system was proposed by Shi et al. (2020) showing that depth data play an important role in a grasp pattern definition. Adopting bimodal data scheme, grayscale, and depth information, they improved in 12% the classification accuracy using four types of grasp patterns, named tripod, cylindrical, lateral, and spherical. A specific dataset was built using Kinect 2.0 with objects of different sizes and shapes. After alignment and filtering, color image with reduced resolution, grayscale images, and depth images (all in 32 × 32) were used as the inputs of the two channels of independent convolutional networks, based on the Cifar-10 model, running on a personal Laptop (Intel Core i5-3210M, 2.5 GHz, 64 bits, Win10) and connected to a prosthetic device. sEMG control was also provided, based on two finite state machines set up to divide the hand control into coding and motion states. The system accuracy was 93.9%, and the tripod grasp was the main misclassification pattern.

As can be seen, image processing systems usually use robust external computers (CPU), which make the application unfeasible for daily living activities context. The best classification rate was 93% achieved by both Shi et al. (2020) and DeGol et al. (2016). The first used a 3D camera providing depth data and two channels of independent convolutional networks based on the Cifar-10 model for four classes (cylindrical, key grasp, spherical, and tripod), while the latter used a 16-layer VGG-VeryDeep-16 convolution neural network for five classes (power grasp, pinch grasp, tool grasp, 3-jaw chuck, and key grasp).

Within this context, a new intelligent hybrid prosthesis model is proposed, commanded by a simple sEMG system aided by a fully embedded CV system. A modified 5-layer SmallerVGG convolutional neural network classifies objects regarding the hand gestures used to interact with them without explicitly identifying them. The system offers five modes: palmar grasp with the wrist in a neutral position and with the wrist pronated, tripod pinch, key grasp, and the index finger extension gesture. This intelligent model facilitates and will speed up the process of learning and using the prosthesis.

## 2. Materials and Methods

### 2.1. System Design

**Figure 1:**
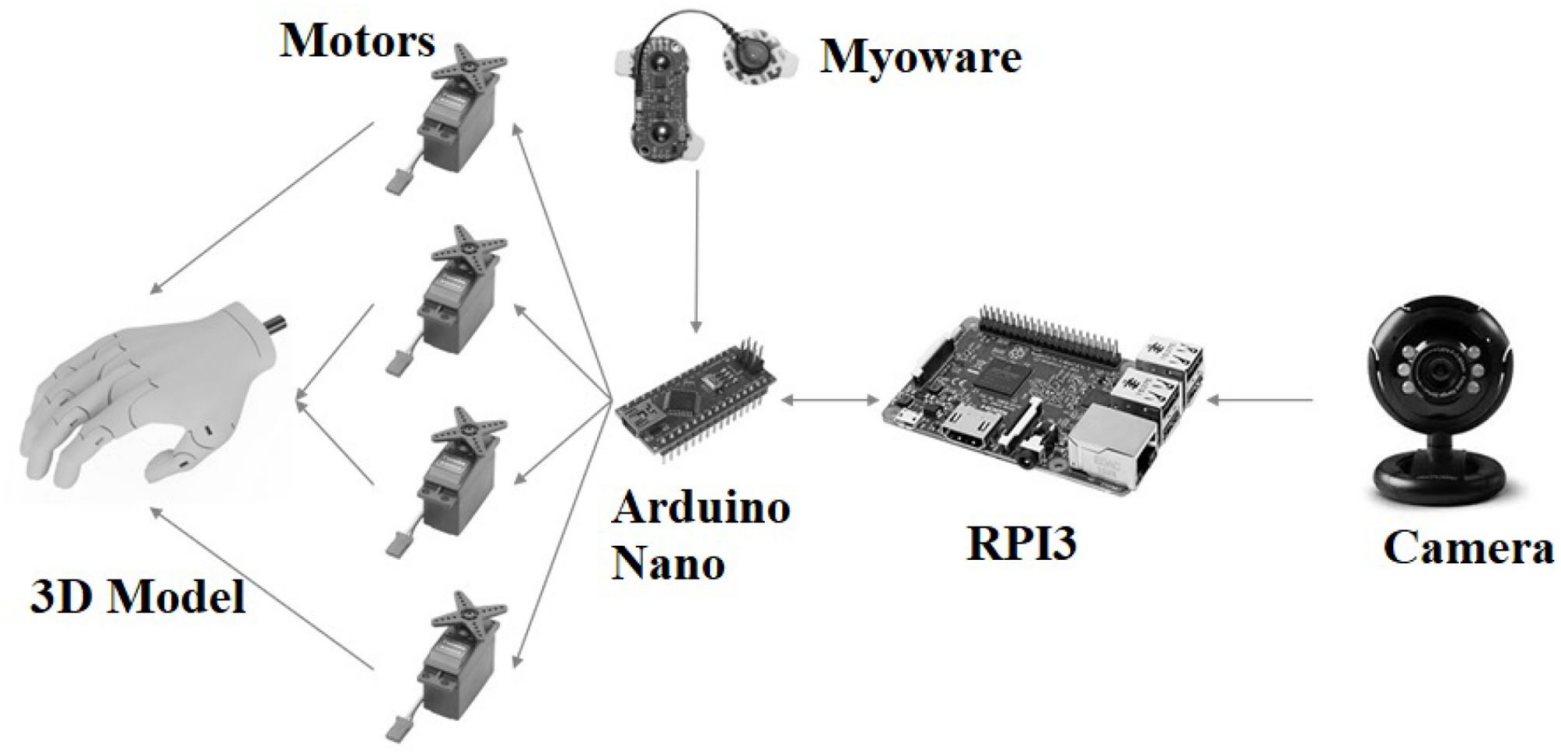
Prosthesis prototype diagram system.

The prosthesis prototype system (Figure 1) is composed of a 3D printed model, an Arduino Nano board, a Myoware sEMG system, a CV system, and an RPI3. The 3D model was based on Buchanan's Kwawu Arm 2.0 and printed on lactic polyacid (PLA). The Arduino system commands the start of image processing, opening, and closing of the prosthesis using servo motors, based on the user's intention detected by the sEMG system and a finite state machine. The CV system is responsible for capturing the image of the object the user wants to grasp, and a CNN, running on the RPI3, classifies it according to the five hand posture patterns.

**Figure 2:**
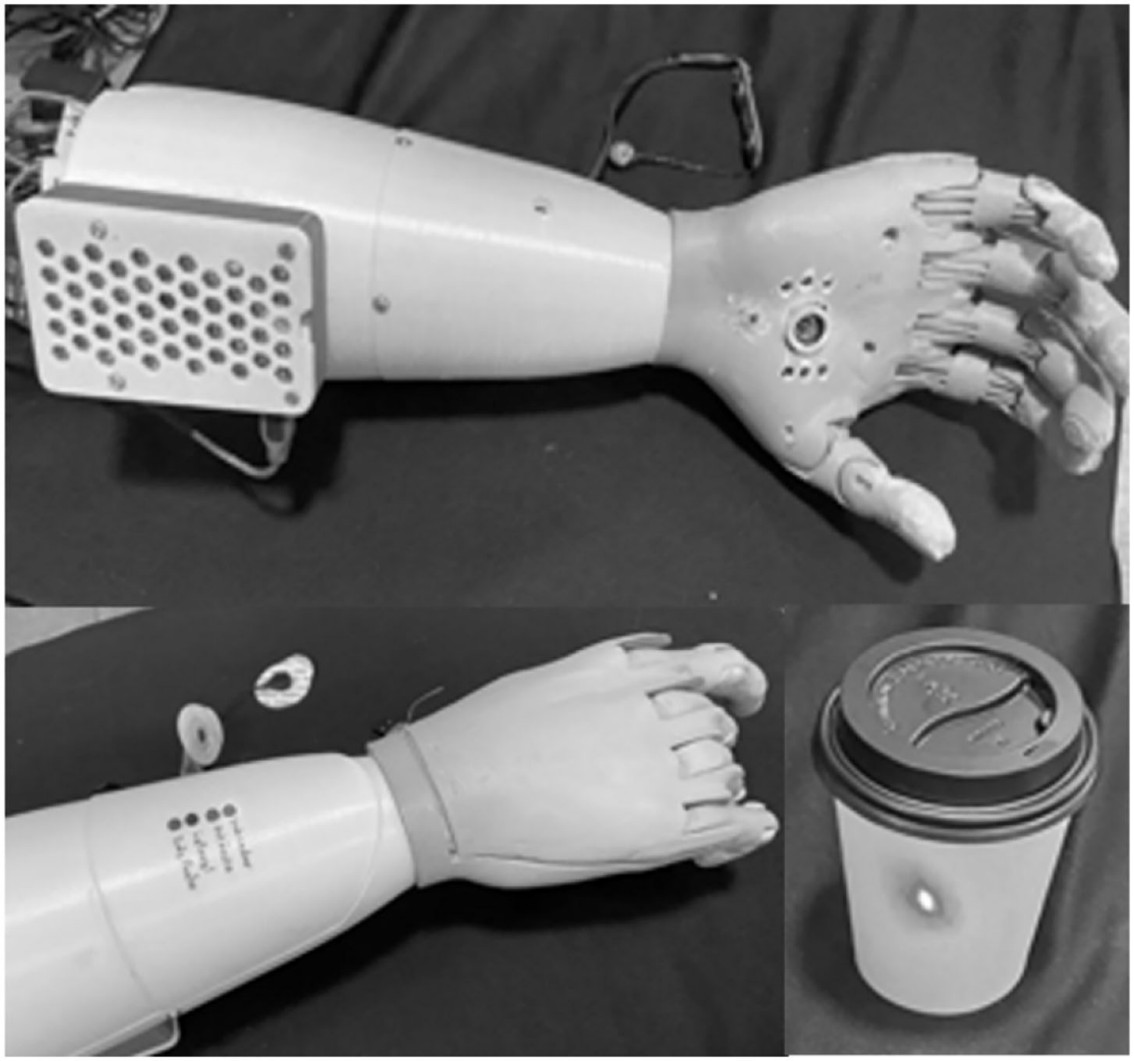
3D prosthesis prototype with the camera, laser point, and LEDs.

The 3D prosthesis model was modified after being printed for the camera, laser point, and LEDs installation. A USB camera, APP-TECH model of 16 Megapixels, and a laser point indicator were installed in the palm area, as shown in Figure 2. The laser point indicates the object to be photographed and handled by the user. LEDs on the back of the prosthesis prototype inform which grasp class was proposed by the neural classifier.

### 2.2. Control System

The sEMG system is comprised of the Myoware Muscle Sensor from Advancer Technologies. It is an Arduino-powered all-in-one sEMG board with adjustable gain, providing the raw and the envelope of the filtered and rectified signal. In this application, the latter was used. The wearable design allows the disposable electrode attachment directly to the board through embedded electrode connectors. Adhesive disposable electrodes from Meditrace were used to capture sEMG signals. The Arduino Nano board, which makes the analog/digital conversion, has a sampling rate of 9,600 samples per second and 10 bits of resolution.

**Figure 3:**
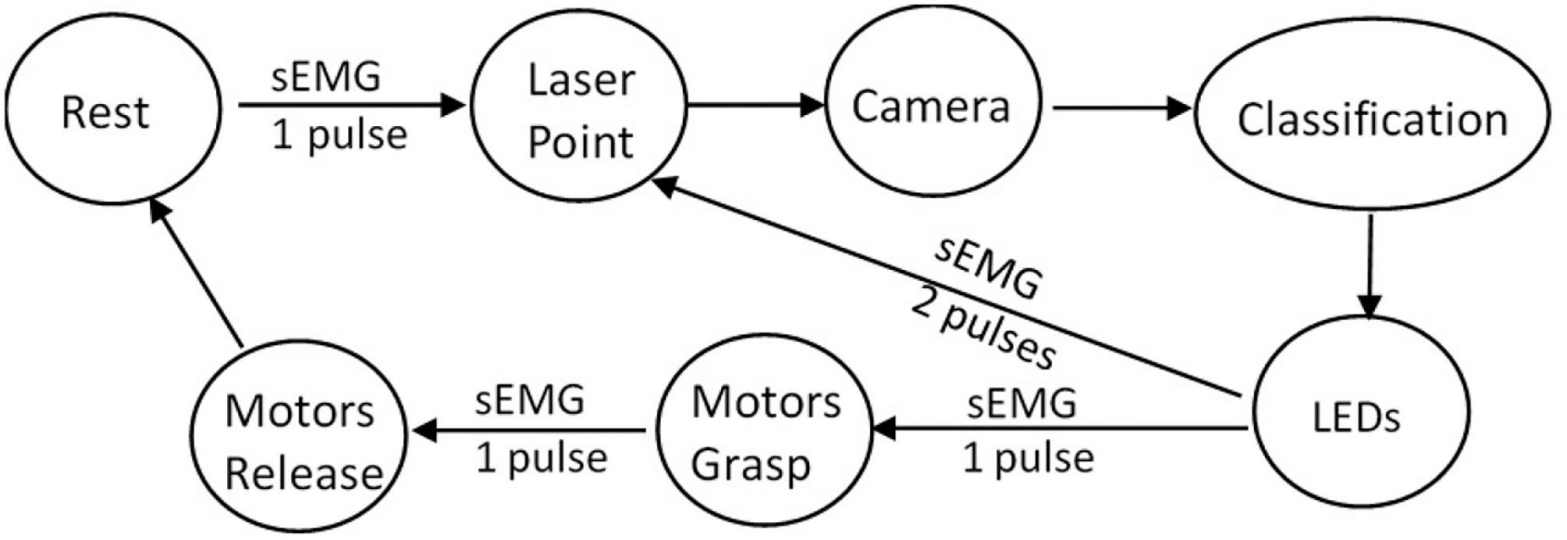
Finite state machine diagram.

The control system is expressed by a finite state machine, which diagram can be seen in Figure 3. For each supra threshold muscle contraction, the control system receives an input pulse. A muscle contraction activates the laser point, so the user visually confirms the object to be picked up, photographs it, and starts the classification process by the CNN. The pattern chosen by the neural network is displayed on the LEDs on the back of the prosthesis. The user has two options: reject and restart the process or accept and command the movement. In the latter, another muscle contraction defines the object release, and the prosthesis returns to its initial condition.

The total estimated time for this state machine to grasp the object since rest is 1.4 s, excluding the time the user takes to accept the suggested grasp pattern. The estimated time for each sEMG pulse is 100 ms, the laser point takes 350 ms, the classification time since camera activation was less than 250 ms, and the time for motor activation and movement was approximately 600 ms.

A 5-layer VGG network (Rosebrock, 2018a,b), a modified version of the VGG-16 (Simonyan and Zisserman, 2015), was used. The input image with 96 × 96 pixels × 3 channels passes through a 3 × 3 convolution filter, followed by a linear rectified function (RELU) and a normalization function (BATCH). The network's first pooling layer uses a 3 × 3 matrix to reduce image dimensionality to 32 × 32 pixels. In the consecutive layers, the dimension of the convolution filters is changed from 32 to 64 and finally from 64 to 128. In all intermediate layers, the DROPOUT function is applied, which disconnects 25% of the layer's neurons to reduce overfitting. The final layer is fully connected through the DENSE function that uses a linear rectifier activation function and then goes through a SOFTMAX function to return the value of the probability of classification of each class. Keras software was used as an application programming interface and TensorFlow as numerical computing software.

The training and validation phases were conducted on a Mac mini 2012 computer (2.3 GHz, quad-core i7, 16 GB) and the final CNN model run on the RPI3 (1.2 GHz, Quad-Core Boadcom BCM2837, 1 GB, running with the Raspbian system) for the final testing. The algorithm converts the images to grayscale and resizes them to 96 × 96 pixels. All images contain a single object, black background, and were taken with ambient lighting.

### 2.3. Experiments

**Figure 4:**
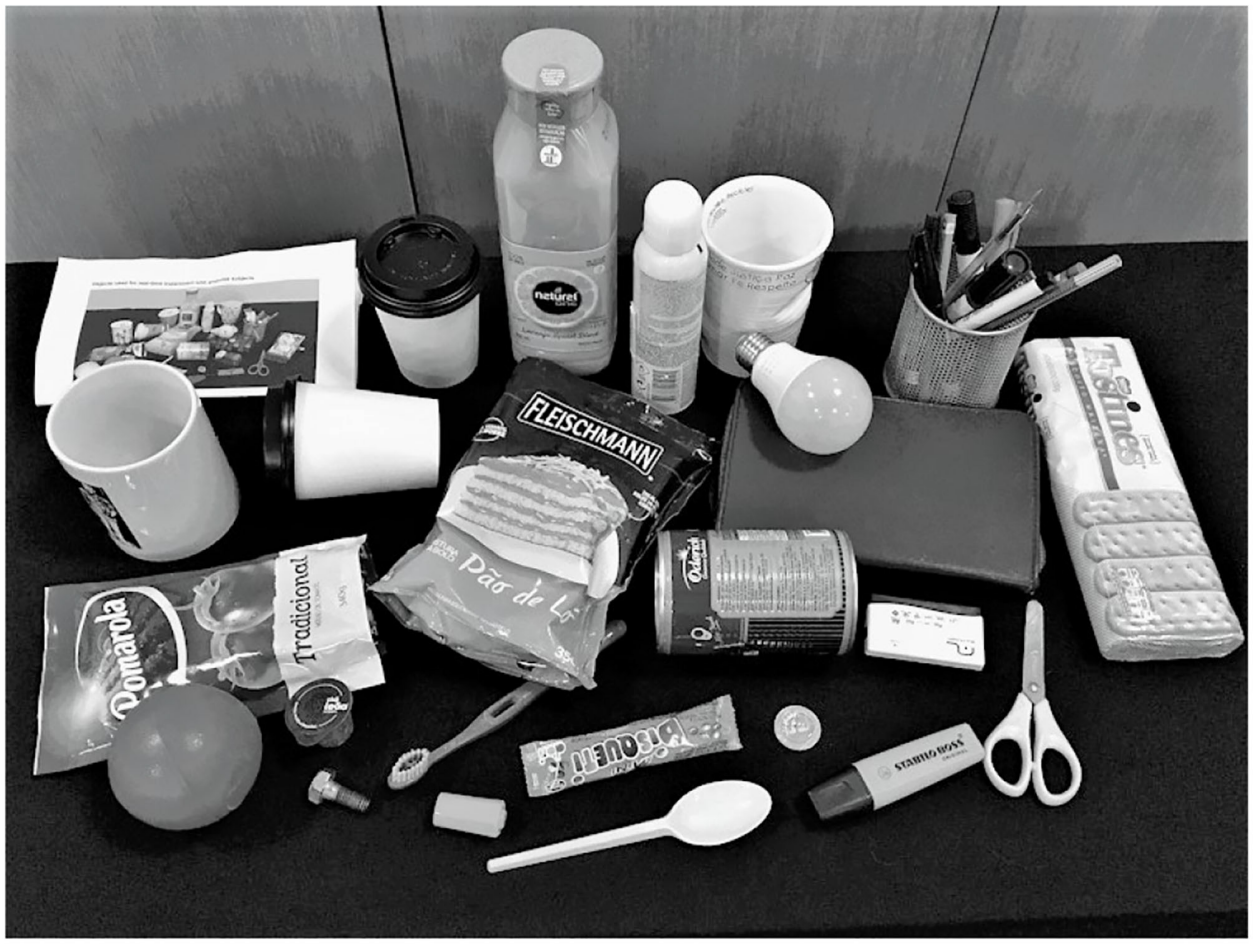
Objects used for comparison between experiments.

Three experiments were carried out using images from the Newcastle Grasp Library (NGL) associated with the Amsterdam Library of Object Images (ALOI), while the final tests were carried out with a set of 24 objects (Figure 4) plus 14 keyboard images, establishing a total of 182 images not presented in the training/validation set. This set of images allowed comparisons of experiment performance.

For experiment 1, the objective was to compare the classification results of the modified 5-layer SmallerVGG network with the experiment of Ghazaei et al. (2017). The training/validation set had 7,632 images for the pinch classification pattern, 11,810 images for the tripod pinch, 8,777 images for the palmar grasp with neutral wrist position (neutral), and 11,304 images for the palmar grasp with pronated wrist (pronated). The 182 images for the test phase had 42 images for the pinch classification pattern, 42 for the tripod, 42 for neutral, and 56 for the pronated.

Due to the experiment 1 results, changes were made to the experiment 2 dataset, eliminating images of objects with similar shapes from different classes, choosing only tripod pinch (between the precision grasps), and adding the key grasp class. The CNN training set had 6,900 images for the tripod pinch, 8,345 images for the neutral pattern, 8,280 images for the pronated, and 2,188 images for the key grasp class. The tests were performed with 70 images for the tripod pinch, 42 for neutral, 56 for pronated, and 14 for the key grasp.

For experiment 3, 8.354 images were added to the experiment 2 dataset, being 3,210 computer keyboard images, 2,808 musical keyboard images, and 2,336 tablet images. Classifications from these three new image classes result in the index finger extension movement. The tests were performed with 182 images being 70 for the tripod pinch pattern, 42 for neutral, 42 for pronated, 14 for key grasp pattern, and 14 for index finger extension.

For the test phases mentioned before, a set of 24 objects, like those used by Ghazaei et al. (2017) (Figure 4), plus 14 images of keyboards and tablets were used, establishing a total of 182 images allowing for result comparisons. Just as Ghazaei et al. (2017), seven images for each object with random angles of view were presented to the classifier.

## 3. Results

Table 1 shows the confusion matrix and Table 2 shows the sensitivity, specificity, and accuracy values, for experiment 1. It can be noted from Table 1 that pinch and pronated patterns had an excellent result, with almost 100% of classification (41 from 42 and 51 from 56, respectively). On the other hand, a huge misclassification (41 from 42 trials) appeared for the tripod pattern that was classified as pinch, and a smaller one appeared for the neutral pattern (11 from 42 trials) that was classified as pronated. Following these results, Table 2 reflect the impact of the right and wrong classifications for each pattern, showing a high sensitivity, but lower specificity for pinch, a very low sensitivity, but high specificity for tripod, a low sensitivity, but high specificity for neutral, and high sensitivity and specificity for pronated.

**Table 1:** Confusion matrix for experiment 1.

	**Pinch**	**Tripod**	**Neutral**	**Pronated**	**Total**
Pinch	41	0	0	1	42
Tripod	41	1	0	0	42
Neutral	0	0	31	11	42
Pronated	3	2	0	51	56
Total	85	3	31	63	182

**Table 2:** Performance metrics of experiment 1 [Se, sensitivity (%); Sp, specificity (%); Acc, accuracy (%)].

	**Se**	**Sp**	**Acc**
Pinch	98	69	75
Tripod	2	99	76
Neutral	74	100	94
Pronated	91	90	91
Total	68	89	84

Table 3 shows the confusion matrix and Table 4 shows the sensitivity, specificity, and accuracy values, for experiment 2. In Table 3, it can be noted that all patterns had an excellent result, with almost 100% of classification, resulting in high sensitivities, specificities, and accuracies up to 96%.

**Table 3:** Confusion matrix for experiment 2.

	**Tripod**	**Neutral**	**Pronated**	**Key grasp**	**Total**
Tripod	68	0	0	2	70
Neutral	0	41	1	0	42
Pronated	0	0	54	2	56
Key grasp	3	0	0	11	14
Total	71	41	55	15	182

**Table 4:** Performance metrics of experiment 2 [Se, sensitivity (%); Sp, specificity (%); Acc, accuracy (%)].

	**Se**	**Sp**	**Acc**
Tripod	97	97	97
Neutral	98	100	99
Pronated	96	99	98
Key grasp	79	98	96
Total	96	99	98

Table 5 shows the confusion matrix and Table 6 shows the sensitivity, specificity, and accuracy values, for experiment 3. In this experiment, despite having one more class, the results of Table 5 showed almost 100% of classification with very low misclassifications, resulting in high sensitivities, specificities, and accuracies up to 98%.

**Table 5:** Confusion matrix for experiment 3.

	**Tripod**	**Neutral**	**Pronated**	**Key grasp**	**Index finger ext**.	**Total**
Tripod	68	0	0	2	0	70
Neutral	0	42	0	0	0	42
Pronated	0	0	41	0	1	42
Key grasp	1	0	0	12	1	14
Index finger ext.	0	0	0	0	14	14
Total	69	42	41	14	16	182

**Table 6:** Performance metrics of experiment 3 [Se, sensitivity (%); Sp, specificity (%); Acc, accuracy (%)].

	**Se**	**Sp**	**Acc**
Tripod	97	99	98
Neutral	100	100	100
Pronated	98	100	99
Key grasp	86	99	98
Index finger extension	100	99	99
Total	97	99	99

## 4. Discussion

**Figure 5:**
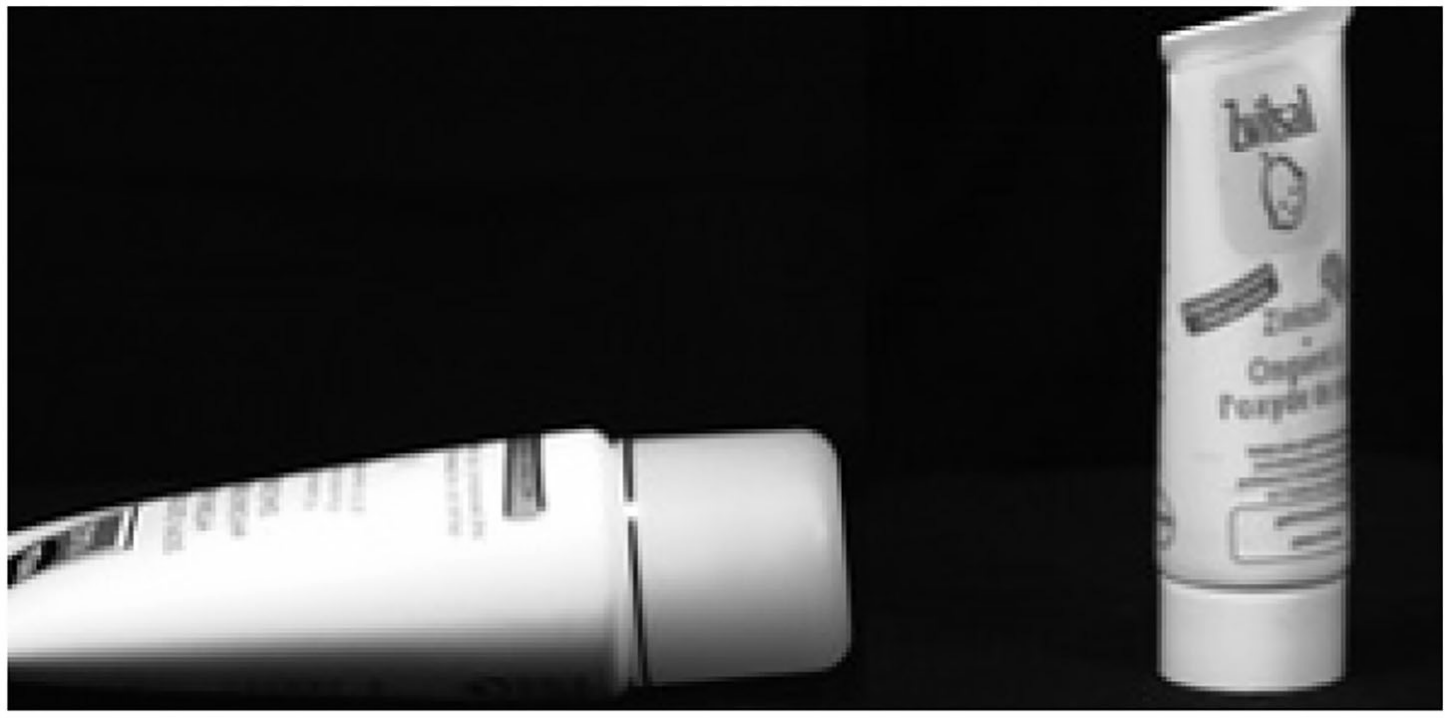
Different tube positions allowing the use of different grasps, such as pronated palmar, tripod, pinch, and neutral palmar.

Defining the type of grasp to pick up objects is not as easy as it may seem because there is no consensus. Even with studies like Feix et al. (2016) and Abbasi et al. (2019), which propose grasp taxonomies, the number of grasp patterns is impractical in such type of system. Furthermore, it is reasonable to pick up an object in different ways, whether it is in the same position or if it is arranged in different orientations, as shown in Figure 5. Thus, it is often a matter of convention.

**Figure 6:**
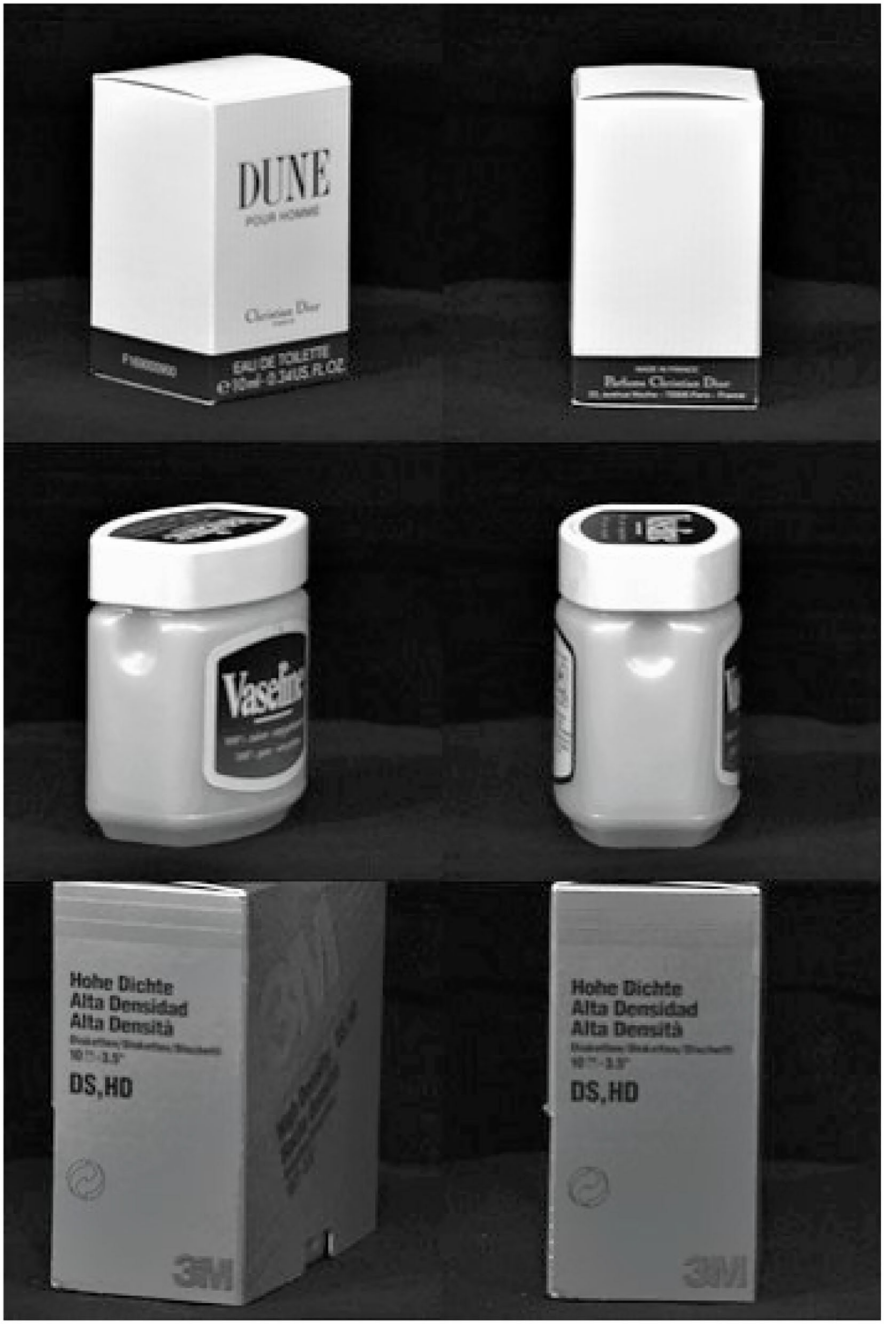
Objects with similar shapes placed in different classes: first line neutral palmar, second and third lines pronated palmar.

**Figure 7:**
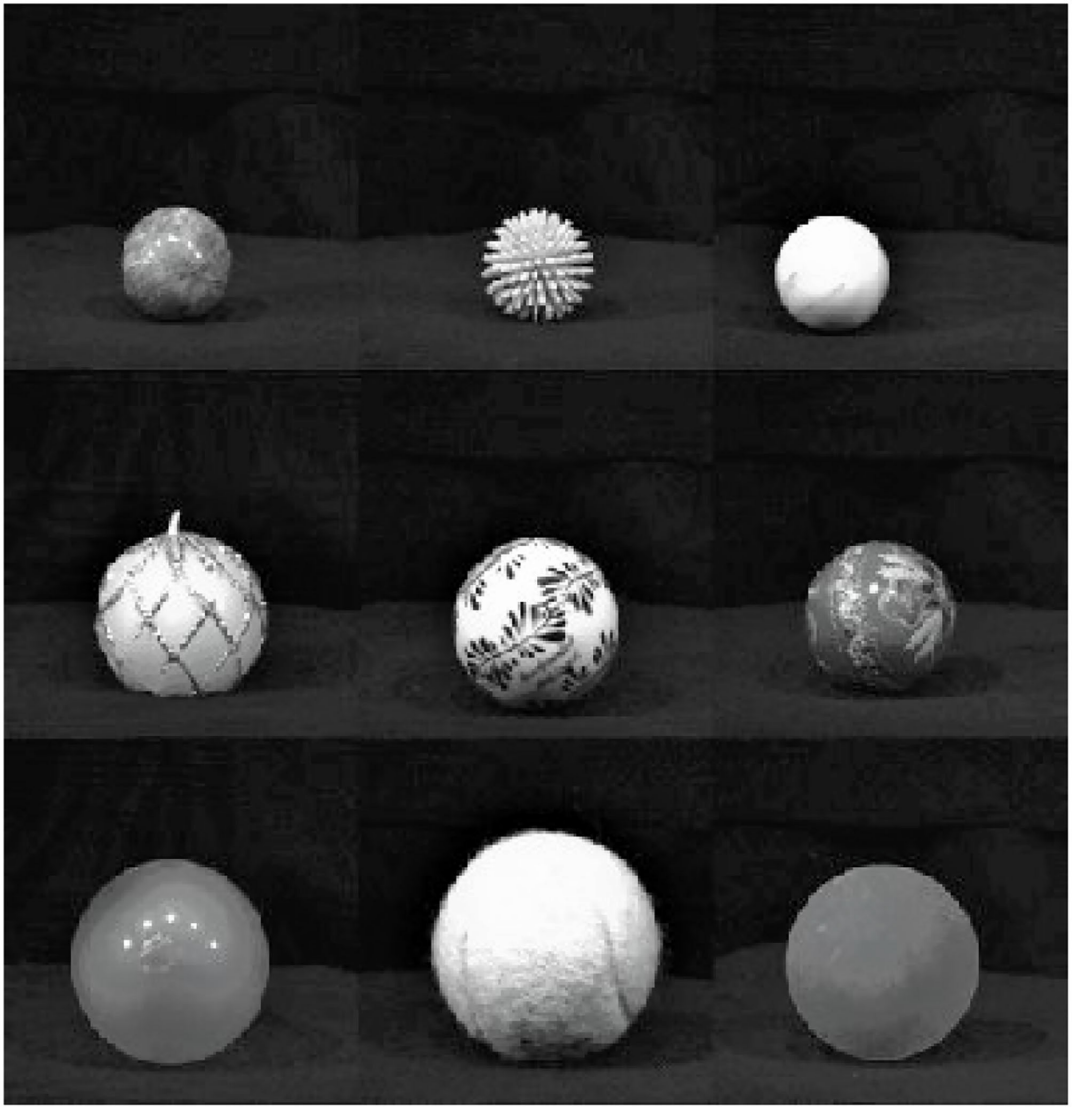
Objects with similar shapes placed in different classes: pinch, tripod, and pronated palmar, respectively.

The proposed 5-layer SmallerVGG trained in experiment 1, with the same image dataset as Ghazaei et al. (2017), achieved an accuracy of 84%, with new objects, as shown in Table 2. The same result was reached by the original work with new and seen images but randomly-rotated objects. The misclassifications, shown in Table 1, resulted in a high sensitivity but low specificity for pinch, a very low sensitivity but high specificity for tripod, and a low sensitivity by but high specificity for neutral (Table 2). Sensitivity is a measure of how well a test can identify true positives, and specificity is a measure of how well a test can identify true negatives. The overall result for experiment 1 was sensitivity equal to 68% and specificity equal to 89%, which will frustrate the user and make the system non-functional. Since the classification feature was based on object shapes, the explanation of these misclassifications was the arrangement of objects with similar shapes in different classes. Clear examples can be seen in Figures 6 and 7. Figure 6 shows objects with rectangular shape, and despite the possibility of using both neutral and pronated palmar grasps to pick them, as the system use the shape as a classification feature, it is not reasonable to have them into different classes. The same situation occurs for the balls with different sizes of Figure 7.

In order to resolve this incompatibility and have a better model, changes were made to the experiment 2 dataset, eliminating images of objects with similar shapes of different classes, choosing only tripod pinch between the precision grasps, since it covers both types of objects, and adding the key grasp class due to its importance, according to Feix et al. (2016) and aiming to keep the same number of classes. These modifications improved the accuracy to 98%, sensitivity to 96%, and specificity to 99% as shown in Table 4, for experiment 2, proving the fragility of the original image bank.

In experiment 3, the proposed 5-layer CNN trained with the modified image bank added with the extension index class obtained 99% of accuracy, 97% of sensitivity, and 99% of specificity as shown in Table 6. It can be said that the proposed CNN, trained for the neutral and pronated palmar grasps, tripod pinch, and key grasp, recognizes patterns, while for the computer and music keyboards, and tablet classes, the network recognize objects. Even with only five convolutional layers, the SmallerVGG network showed high accuracy in classifying these patterns and objects in a hybrid configuration.

Comparing the sensitivity and specificity values, experiments 2 and 3 showed greater effectiveness in classification than experiment 1, due to the consistency of the image dataset, presenting a smaller number of false-negative and false-positive results, showing the importance of the adequacy of the image set for the success of the classification. These results contribute to user satisfaction and system functionality.

Compared with other works in the literature that used a CV system to define the grasp patterns for the prosthesis, the proposed system with a 5-layer SmallerVGG CNN achieved an accuracy higher than those proposed by DeGol et al. (2016) and Shi et al. (2020) that presented accuracies of 93% with a bimodal data scheme and a VGG-VeryDeep-16, respectively.

Using an RPI 3 microcomputer instead of a computer for real-time analysis associated with a 3D printed model prosthesis turned the project into a low-cost portable prototype. It is a full embedded control system, with higher accuracy than those proposed by Dosen et al. (2010), Dosen and Popovic (2010), Ghazaei et al. (2017), and Shi et al. (2020) in which the processing was done in a separate unit as a standard PC.

Moreover, the proposed hand prosthesis prototype focused on a more natural appearance, incorporating a discreet webcam in the palm, unlike the proposals by DeGol et al. (2016), Ghazaei et al. (2017), Dosen et al. (2010), and Dosen and Popovic (2010) who used an external webcam. The Arduino Nano board used to command the servos can be substituted, as the RPI3 can perform this task. This change would reduce the internal wires and cables and increase the space available inside the prosthesis prototype body.

The Myoware sensor board has a gain adjustment of the sEMG signal, and the software has a threshold adjustment, which allows for the customization required for each user due to the sEMG electrode positioning and physical conditions. Despite its simplicity, it seems to be enough to the proposed application of state machine trigger, instead of a more sophisticated one such as Myo armband as used by Andrade et al. (2017) or Delsys Trigno used by Ghazaei et al. (2017).

The total estimated time for the state machine to grasp the object since rest is approximately 1.4 s, which is reasonable for prosthetic control. However, the time taken for the user to confirm the grasp pattern was not considered. The time for each sEMG pulse was estimated at 100 ms, and it depends on the user's ability to fast contract above the selected threshold. The laser point of 350 ms aims only to confirm the selected object to be grasped. The classification time since camera activation was approximately 250 ms, and the time for motor activation and movement was approximately 600 ms.

Ghazaei et al. (2017) reported 150 ms for the average time needed for pre-processing and classification in computer-based real-time performance analysis, and 300 ms using a short flexion contraction above a threshold. Sidher (2017) reported classification times varying from 223 ms to 1.963 s for geometrical objects using RPI 3. Compared with the previous studied, the proposed system control presents promising behavior.

On the other hand, the presented prototype did not intend to be a final prosthesis proposal but a proof of concept of the feasibility of a fully embedded hybrid system based on a hybrid approach using sEMG and CV to overcome the limitations of the strict sEMG control systems. Therefore, the experiments reported in this study were related to the CV technical aspects.

Some improvements could be performed, such as adding new classes to the CV classification system or some common gestures as a user choice option via the sEMG finite state machine. Examples of the first are parallel extension, hook, and power grip, and for the latter are point (index finger extension), ok sign, and thumbs up.

Furthermore, the results were obtained in a controlled environment, with fixed prototype distance and height related to the object. These parameters could change the classification accuracies due to the classifier model's dependence on object shape patterns. Adding distance sensors like Dosen et al. (2010), Dosen and Popovic (2010), and Sidher (2017) did, seem reasonable to overcome the problem of similar shape but different size objects, as shown in Figure 7.

Therefore, the prosthesis's functional performance evaluation in a clinical trial is essential to guarantee its effectiveness. The Southampton Hand Assessment Procedure (SHAP) is a well-known, simple, and replicable protocol based on the assessment of the effectiveness of the prosthetic device with a focus on performing a set of tasks by the user (Light et al., 2002; Andres-Esperanza et al., in press). Dosen et al. (2010) showed that the average time to accomplish the “reach, pick up and place” task with 13 healthy subjects decreases with training, reaching approximately 10 s after 100 trials. Shi et al. (2020) reported an average time of 6.4 s in an experimental protocol with four healthy subjects performing a total of 320 trials, comparing Vision-EMG and Coding-EMG control. Ghazaei et al. (2017) reported an average time of 7s for two trans-radial amputee volunteers to accomplish the “reach, pick up, and place” task. However, this evaluation is not the focus of this study and will be the subject of future investigation.

## 5. Conclusion

This study presented a hybrid 3D printed hand prosthesis prototype based on an sEMG controlled finite state machine and a fully embedded CV system. A modified 5-layer Smaller Visual Geometry Group (VGG) CNN running on an RPi 3 connected to a webcam recognizes the shape of daily use objects and defines the grasp/gesture pattern for the prosthetic prototype. The sEMG signal, representing the user's intention, starts the process and commands the prosthetic motors to movement execution.

The proposed system obtained 99% accuracy, 97% sensitivity, and 99% specificity for grasping objects from neutral and pronated palmar grasp, tripod pinch, key grasp, and index finger extension gesture. Compared with other studies in the literature that used a CV system for prosthetics, the proposed system achieved a higher accuracy with a full embedded system. Furthermore, it is a low-cost technology with a reduced user training time, considering the simple use of sEMG.

This study showed that the use of a vision system to help define the pattern of grasping and manipulating objects is a promising alternative and that studies in this area should be performed. For the continuity of this study, it is proposed the improvement of the prosthesis for thumb movement; prosthetic functional performance evaluation in clinical measurements to guarantee its effectiveness.

## Data Availability Statement

The raw data supporting the conclusions of this article is available at: https://doi.org/10.5281/zenodo.5749745.

## Author Contributions

GR was the engineer and developer of all presented platforms. MC masterfully coordinated the work and was a major contributor in writing the manuscript. WP contributed to reviewing the manuscript. All authors read and approved the final manuscript.

## Funding

This study was financed in part by the Coordenação de Aperfeiçoamento de Pessoal de Nível Superior—Brazil (CAPES)—Finance Code 001.

## Conflict of Interest

The authors declare that the research was conducted in the absence of any commercial or financial relationships that could be construed as a potential conflict of interest.

## Publisher's Note

All claims expressed in this article are solely those of the authors and do not necessarily represent those of their affiliated organizations, or those of the publisher, the editors and the reviewers. Any product that may be evaluated in this article, or claim that may be made by its manufacturer, is not guaranteed or endorsed by the publisher.
